# Diagnostic accuracy of the international classification of disease “I26” code to detect acute pulmonary embolism in a surveillance network

**DOI:** 10.1016/j.rpth.2025.102900

**Published:** 2025-05-23

**Authors:** Jeffrey A. Kline, Jesse O. Wrenn, Mazin F. Alam, Alexis N. Drinkhorn, Conner D. Slotnick, Fawas Shaman, Christopher E. Conn, Steven J. Korzeniewski, Christopher Kabrhel

**Affiliations:** 1 Department of Emergency Medicine, Wayne State University, Detroit, Michigan, USA; 2 Department of Emergency Medicine, Vanderbilt University Medical Center, Nashville, Tennessee, USA; 3 Department of Family Medicine and Public Health Sciences, Detroit, Michigan, USA; 4 Department of Emergency Medicine, Massachusetts General Hospital, Harvard Medical School, Boston, Massachusetts, USA

**Keywords:** diagnosis, emergency medicine, electronic health records, epidemiology, pulmonary embolism, surveillance

## Abstract

**Background:**

Emergency departments (EDs) offer a unique platform for a surveillance network for acute pulmonary embolism (PE) using International Classification of Disease (ICD-10) codes extracted from electronic medical records.

**Objectives:**

Test the diagnostic accuracy of the I26 "leader" ICD-10 code for the detection of PE in near real-time in a large, ED-based surveillance network.

**Methods:**

Standardized structured language queries were deployed at 91 hospitals to extract data, including ICD-10 codes, on a weekly basis from electronic medical records on ED patients with acute respiratory complaints. We used 2 methods for coding computed tomography pulmonary angiogram (CTPA) reports to derive a criterion or gold standard for PE diagnosis: (1) research associates were trained to interpret the CTPA reports, and (2) a validated Regular Expression computer program was used to interpret PE on CTPA reports. These 2 methods were independently adjudicated (PE^+^ or PE^−^). The primary outcome was diagnostic accuracy of the I26 leader compared with the final adjudication.

**Results:**

From 6448 valid CTPA scan reports, 442 (6.8%) were adjudicated as PE^+^. On a weekly basis, the I26 leader had a sensitivity of 50.9% (95% CI, 46.1%-55.6%) and a specificity of 99.7% (95% CI, 99.5%-99.8%), likelihood ratio (LR) negative of 0.49 (95% CI, 0.44-0.54) and LR positive of 191 (95% CI, 116-12). At 1 month, the I26 sensitivity was 57.5% (95% CI, 52.7%-62.1%), and specificity was 99.5% (95% CI, 99.2%-99.6%); LRnegative of 0.43 (95% CI, 0.38-0.47) and LRpositive of 111 (95% CI, 77-159).

**Conclusion:**

For low-latency surveillance of PE diagnosed in EDs, the ICD leader code I26 affords high specificity and high LR(+) for detection of acute PE in the United States but has modest sensitivity.

## Essentials

• No national surveillance system exists for real-time surveillance of venous thromboembolism (VTE).
• An existing network designed for surveillance of acute respiratory illness emergency departments.
• The International Classification of Disease “I26” code demonstrated high specificity for VTE.
• The International Classification of Disease code I26 holds promise as a method for low-latency surveillance of VTE.

## 1 Introduction

Approximately 1 million Americans experience new or recurrent acute pulmonary embolism (PE) each year and approximately 50,000 die, making PE the third most common cause of cardiovascular disease–related mortality [[1], [2], [3], [4]]. Evidence from previous studies raises concern about racial and geographic disparities in care [[1], [2], [3]]. However, existing information from static databases that often report on retrospective data with years of latency experience many limitations, including unknowns about underlying missingness [5]. It is widely believed that at least half of new or recurrent PE cases are diagnosed in an emergency department (ED) setting [6]. Researchers, advocacy groups, and government officials have expressed interest in establishing an ED-based surveillance network for PE [[7], [8], [9]]; namely, because EDs are the largest source of information about people seeking unscheduled acute medical care in the US general population [10].

The frequency of PE may vary by geography, seasonality, and exposure to environmental factors possibly including viral endemics and epidemics [[11], [12], [13], [14], [15]]. However, the dearth of active US surveillance data limits scientific capacity to probe these possibilities. Public health surveillance is the ongoing and systematic collection, analysis, and interpretation of health data in the process of describing and monitoring health events [16]. To meet the criterion of ongoing thus requires a systematic approach to be performed on a preplanned schedule. In the ED setting, this is most likely to occur by automated, computational assessment of information from electronic medical records (EMRs), as demonstrated for other conditions [17]. The identification of ED patients with acute PE (either new or recurrent) ostensibly requires the use of International Classification of Disease 10th edition (ICD-10) codes, notably the I26X cluster, referred to hereafter as the I26 leader. However, the limited diagnostic accuracy of ICD codes in EMRs has been recognized [18]. A systematic review that compared ICD codes against a human-based reference standard found that sensitivity ranged from 20% to 100% with a pooled sensitivity of 72% (95% CI, 60%-85%), while specificity ranged from 63% to 100% with a pooled specificity of 82% (95% CI, 76%-88%) [19]. Unlike a surveillance system, these estimates were produced by retrospective cohort studies representing only cross-sectional points in time. In this work, we examined ICD code diagnostic performance for identifying PE using data collected in real-time on a weekly basis from the Respiratory Virus Laboratory Emergency Department Network Surveillance (RESP-LENS) program contracted by the Centers for Disease Control (CDC) from October 2021 to September 2024 [20].

## 2 Methods

### 2.1 Study design, data, and participants

This cohort study includes information collected by the RESP-LENS network using methods that were described previously [21]. Briefly, RESP-LENS involves the collaboration of 24 investigators representing 91 hospitals who were contracted by the CDC to prospectively surveil viral infections (https://www.cdc.gov/surveillance/resp-lens/dashboard.html). A unique aspect of RESP-LENS was that it established a system and method to extract patient data from hospital EMRs for patients with 1 or more of 130 qualifying ICD codes defining acute respiratory illness (ARI) from the 91 hospitals on a weekly basis with 30-day follow-up. Rationale for these 2 time points is to address the question of whether in real-time surveillance, ICD accuracy changes substantially over a 4-week period. This question may be important toward expectations for frequency of reporting of future surveillance networks.

The network began in September 2021 with the final 24 sites covering all 10 US Department of Health and Human Services regions reporting data as of April 2022 (see Acknowledgments for site information). The protocol was reviewed by all site institutional review boards and was deemed nonhuman subjects research (ie, surveillance) and exempted from review or approved with expedited review.

### 2.2 Data collection procedure

Data were collected for patients with ARI at 2 times; first, from the qualifying index ED visit and then follow-up data were collected again 30 days later. If the patient was discharged on the index visit and had no further encounters, then no new 30-day data were reported. For RESP-LENS, criteria for ARI was defined as having 1 or more of the 130 ICD-10 diagnosis codes that was were derived by consensus among the investigators and CDC personnel (Supplementary File). Many of these ICD-10 codes included descriptive diagnoses that were consistent with symptoms of acute PE. Patients were eligible to be enrolled more than once, and unique patients with multiple visits were identified using their hashed medical record number. Collected data were machine verified for completeness and formatting before upload and then reviewed weekly manual review by 3 CDC analysts for potential anomalies (eg, unexpected changes in testing frequency). As needed, coding updates were made on a weekly basis to account for changes in test ordering patterns. A subset of patients with ARI underwent CPTA scanning, ordered from the ED, as part of usual care.

### 2.3 Extraction of ICD codes

Each patient was identified by weekly electronic query, performed on Sundays, applied to the total ED volume (ie, all) patients who signed into 1 of the 91 EDs participating in RESP-LENS in the previous 7 days. We refer to this collection time point as weekly surveillance. Specifically, the queries (coded in structured query language) identified patients who had 1 or more qualifying ARI-defining ICD code that was recorded in the ED record of the EMR at the time of ED discharge or hospital admission. At all sites, data were extracted from either the Cerner Electronic Data Warehouse or the Epic Clarity database. The entire list of ICD codes associated with the index ED visit for patients with ARI were imported each Sunday, and then again on an automated follow-up, 30 days later, referred to as monthly surveillance. The ICD codes examined in this report were those used directly for billing for the ED visit. The purpose of this 30-day repeated assessment is to address inherent latency in ICD coding secondary to human factor delays in charting billing and coding, and the fact that some patients who registered near the end of the week (Saturday or Sunday) may not have had medical records completed by the time data were uploaded. The RESP-LENS database also required upload of all charge procedure terminology codes at index and within 30 days.

This analysis of RESP-LENS data examines the diagnostic accuracy of the ICD I26 leader who presented to the EDs compared with uploaded final radiologist reports from computed tomography pulmonary angiography (CTPA) as the criterion standard. All RESP-LENS sites were encouraged to upload CTPA reports, but only a subset of 44 hospitals in 9 states (Colorado, Massachusetts, Michigan, Mississippi, Ohio, Oregon, California, Iowa, and West Virginia) uploaded the information because it was not a mandatory field for payment per contract. All CTPA scans that were identified by an order tag as deliberately ordered for PE diagnosis and interpreted by a board-certified radiologist with full transcripts available for analysis were eligible for inclusion. Because RESP-LENS also required upload of all charge procedure terminology codes at index and within 30 days, we additionally queried the CPT codes for ventilation–perfusion (VQ) lung scanning (78,582 and 78,598) to verify the fitness of CTPA as the sole method of PE adjudication.

### 2.4 Criterion standard for PE

We examined the utility of the ICD I26 leader coding collected during the first week and separately at 1 month later to detect acute or chronic PE compared against the combined adjudication outcome of 2 criterion (ie, gold) standards: one being the interpretation of CTPA by 5 trained research associates and the other involving the use of a previously validated computerized algorithm for Regular Expression Aided Determination of PE (READ-PE) from CTPA reports [22].

#### 2.4.1 Human adjudication

Five research associates with previous research experience were trained by the senior investigator (J.A.K.) on how to interpret the radiologists’ CTPA reports during 3 separate 2-hour sessions. The research associates performed several practice sessions on samples of 100 that were previously coded by the senior investigator, followed by a debriefing and education session focused on any indeterminate, false-positive, or false-negative interpretations. The adjudicators were taught to interpret words in the radiologists’ reports to distinguish findings of acute PE vs chronic PE and either finding was considered positive. They were also taught to code for features of the scan suggesting an indeterminant result, based upon radiologist mention of inadequate pulmonary arterial opacification, or motion artifact. However, for this report, we decided a priori to code those with indeterminant features as negative for PE. Each of the 5 adjudicators were then assigned a random sample of 1400 unique cases to be interpreted within 2 months. The adjudication results were recorded using a REDCap (www.projectredcap.org) survey shown in Supplementary File.

#### 2.4.2 Computer-assisted adjudication

The same set of CTPA reports used for human interpretation were used for computerized interpretation. The CPTA reports were independently analyzed using the previously validated READ-PE algorithm was demonstrated to have >93% sensitivity and 99% specificity for PE detection [22]. The READ-PE algorithm involves structured query language that combines a set of regular expressions with simple logic to identify the presence of PE in the impression section of CTPA reports. In brief, text is divided into individual sentences using the period (‘.’) character that are evaluated across a set of regular expressions to identify phrases indicating positivity for PE and phrases indicating negation or an alternative to PE. If any sentence in the report has a match for a positive phrase without any negative phrases, then the CTPA report is considered positive for PE. The READ-PE only interprets a binary result (yes or no PE) and does not interpret scans as indeterminant. For this project, READ-PE was adapted to evaluate full heterogeneous radiology reports instead of the radiology interpretation on which it was derived and externally validated by excluding sections and language describing patient history and CTPA indication and protocol. The output of READ-PE was compared against the human adjudicator responses, and discrepancies were resolved on a case-by-case basis by consensus of senior members of the research team (J.O.W. and J.A.K.) to create a final criterion standard of PE+.

#### 2.4.1 Clinical features of patients

To afford a contextual understanding of the patient population, and to allow comparison of our patient sample to other patient samples in future literature, we also include clinical characteristics that are particularly relevant to the ED setting. These included vital signs, arrival method, demographics, and a medical history. This is also relevant inasmuch as it shows the level of detail that can be extracted on a weekly basis. In addition, we thought it important to determine whether the PE+ and no PE groups differed significantly in in any particular variable.

### 2.5 Statistical analysis

Proportions of clinical features were compared with a chi-squared test between PE+ and no pe patients. The primary analysis focused on the outputs from the 2 × 2 diagnostic contingency table, comparing the ICD26 leader with the criterion standard, including sensitivity, specificity, likelihood ratios, and positive and negative predictive values with 95% CIs, calculated from the exact Clopper–Pearson method. We also compared the hospital-to-hospital variation of diagnostic accuracy of the ICD26 leader from hospitals that had >100 CTPA scans fully adjudicated. The rationale for the 100 cutoff is that samples <100 often contained very few or no PE-positive cases. We also tested for correlation between the likelihood ratio positive and CTPA volume and ED volume after confirming normality (Shapiro–Wilk *P* > .1) using Pearson correlation coefficient with 95% CIs from Fisher z transformed (Stats Direct 4.04). Statistical differences were determined based on nonoverlapping 95% CIs. RESP-LENS adheres to the STROBE (Strengthening the Reporting of Observational Studies in Epidemiology) and RECORD (Reporting of studies Conducted using Observational Routinely-collected Data) reporting guidelines [23,24]. The calculated sample size of 7000 was predicated on the assumption that approximately 10% of scans would be excluded (ie, either not a CT scan of the chest or not having intravenous contrast) and that at least 6% of the remainder would be positive, yielding approximately 350 PE+ cases, which would allow narrowing of the 95% CI for diagnostic sensitivity to less than ±5% [25].

## 3 Results

Figure shows the flow diagram of all patients surveilled from November 7, 2021, until August 10, 2024 by the 44 sites that contributed CTPA reports to RESP-LENS. During the same period, CPT codes of 14 VQ scans were uploaded.

**Figure:**
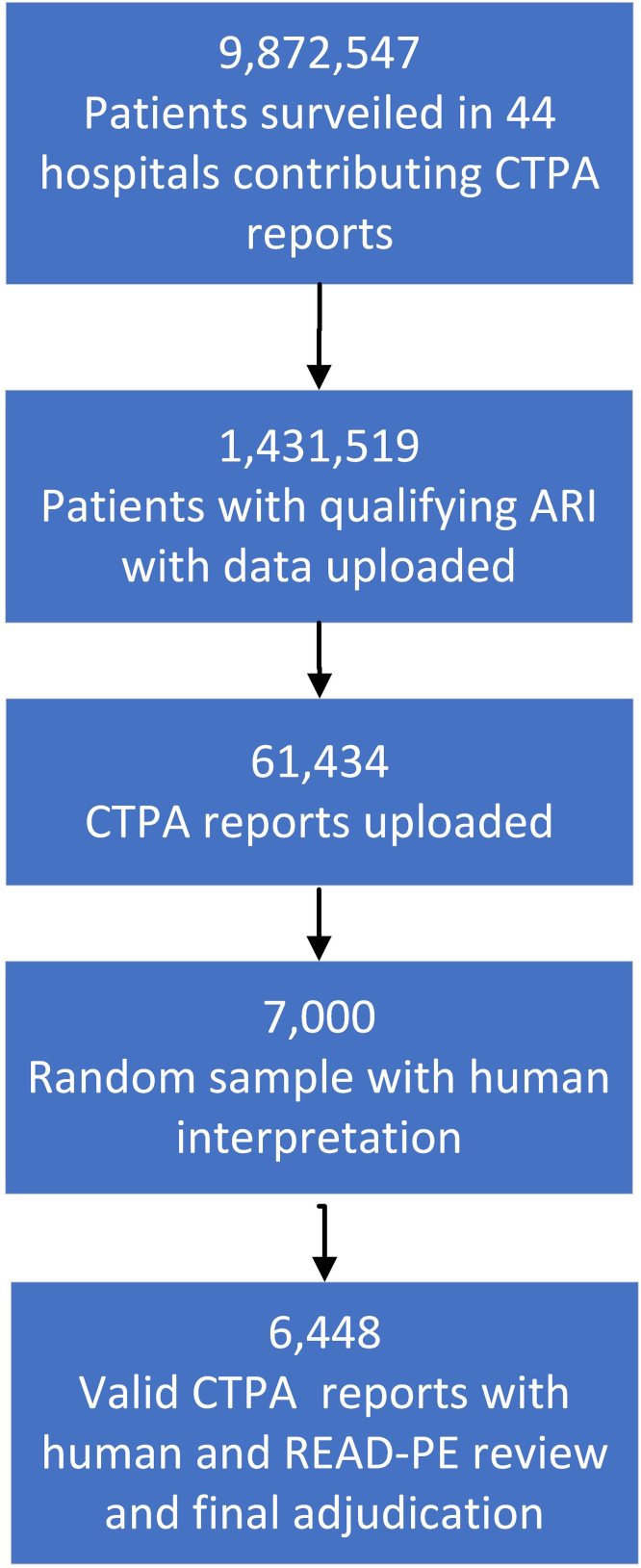
Flow diagram showing how the study population (*N* = 6448) was derived. ARI, acute respiratory illness; CTPA, computed tomography pulmonary angiogram; READ-PE, Regular Expression Aided Determination of pulmonary embolism.

During this time frame of surveillance, the results of 61,434 CTPA scans were uploaded into the RESP-LENS database. The human adjudicators reviewed 7000 radiology reports but excluded 553 because the scan was coded as a CPTA but lacked intravenous contrast. Thus, the final sample size of valid CTPAs was 6448. Of these, 6241 were unique patients (ie, 207 had a repeat ED visit with a qualifying ARI and another CTPA within 30 days of index visit). Of the 6448 scan reports, humans initially adjudicated 472 as PE+. However, when READ-PE algorithm was applied, it found no PE in 44 of these 472 cases. On manual evaluation, 2 investigators deemed that 31 were actually true negatives (no PE), representing false-positive designations in the human adjudications. Upon further evaluation, it was discovered that these 31 false-positive cases, had occurred because of a glitch that caused a “no” to flip to a “yes” when the coder scrolled down the REDCap survey, as opposed to a cognitive error interpreting the CTPA reports. The frequency of disagreement between human-adjudicated reports deemed to be negative for PE and the READ-PE algorithm was low with only 7 discordances (READ-PE found PE+ and humans found no PE) of 6002; after final adjudication, 1 of these 7 cases was converted to PE+. After final reconciliation, the criterion standard was positive for PE in 442 cases (all unique patients) and PE was negative in 6006 cases equating to a prevalence of 6.9% (6.3%-7.5%). Among the 442 cases, 399 (90.3%) had evidence of acute PE, and 43 (9.7%) had only evidence of chronic PE.

Table 1 compared the clinical and demographic characteristics between PE+ and no PE groups. These comorbidities were extracted from the respective data warehouse in Cerner or Epic. These data are often used to auto-populate the past medical history section of templated medical records. The groups were relatively similar in features with the expected exception of prior PE, which was more common in patients with PE+ (P < .001, chi-squared test).

**Table 1:** Comparison of features of patients with and without pulmonary embolism.

Feature	PE+ (*n* = 442)		No PE (*n* = 6006)	
	Mean or count	SD or %	Mean or count	SD or %
Age and vital signs				
Age (y)	64	17	62	18
Respiratory rate (breaths/min)	22	6	21	6
Heart rate (beats/min)	101	22	97	22
Systolic blood pressure (mm Hg)	135	27	139	28
Pulse oximetry (%)	93	10	93	10
Demographic features				
Arrival by ambulance	107	24%	1309	22%
American Indian or Alaska native	0	0%	18	0%
Asian	8	2%	113	2%
Black	73	17%	955	16%
Native Hawaiian or other Pacific Islander	0	0%	7	0%
White	317	72%	4294	71%
>1 race	34	8%	547	9%
Latin or Hispanic ethnicity	10	2%	235	4%
Comorbid conditions				
Smoker	82	19%	1278	21%
Alcohol use	63	14%	661	11%
Systemic hypertension	223	50%	3214	54%
Diabetes	46	10%	896	15%
Obesity diagnosed	59	13%	845	14%
Heart failure	57	13%	829	14%
Atrial fibrillation	32	7%	505	8%
Active cancer	67	15%	997	17%
Chronic obstructive pulmonary disease	48	11%	884	15%
Asthma	43	10%	743	12%
Previous PE	84	19%	267	4%

PE, pulmonary embolism.

### 3.1 Diagnostic performance of ICD coding

Among the 442 patients with the criterion standard positive for PE, the download of ICD codes each Sunday yielded 225 ICD codes with the 3 leading digits of I26, equating to a sensitivity of 50.9% (95% CI, 46.1%-55.6%). Among the 6006 patients with CTPA reports adjudicated as no PE, the weekly ICD download yielded 16 false positives, equating to a specificity of 99.7% (95% CI, 99.5%-99.8%). On a weekly extraction basis, the I26 leader produce a likelihood ratio negative (LR[−] = 0.49 [0.44-0.54] and LR[+] = 191 [116-312]). On monthly extraction, among the 442 PE+ patients, 254 had an ICD code of I26, equating to a sensitivity of 57.5% (52.7-62.1) and 31 of the 6006 no PE patients were false positive, equating to a specificity of 99.5% (99.3%-99.7%). On the monthly extraction, the 126 leader produced a LR negative of 0.43 (0.38-0.47) and LR positive of 111 (77-159). At 30 days, the Boolean “Or” addition of the ICD code for deep vein thrombosis (DVT; I82) captured only 1 additional true positive (255 of 442 PE+ cases detected) but increased false-positive rate from 31 to 67 of 6006.

### 3.2 Interhospital variability

Table 2 presents the results of the 30-day ICD26 leader the 17 hospitals for which we abstracted results and performed REGEX crosscheck on more than 100 CTPA scans. This table demonstrates that the LR+ is above 50 in all but 1 hospital. A second observation is modest but statistically significant correlation between the LR(+) and the sample size (Pearson correlation coefficient, *R*= 0.55; 95% CI, 0.1-0.82). The number of CTPA done were proportional to the ED volumes (*R* = 0.77; 95% CI, 0.46-0.92).

**Table 2:** Comparison of the I26 leader diagnostic accuracy between sites.

Site	*n*	Sensitivity	Specificity	LR(−)	LR(+)	ED volume
1	670	0.37	1.00	0.63	113.9	1,633,671
2	459	0.33	1.00	0.67	33.3	985,615
3	271	0.63	0.99	0.37	79.6	378,582
4	204	0.67	0.99	0.34	63.0	138,216
5	410	0.65	1.00	0.35	254.3	312,298
6	202	0.75	1.00	0.25	150.0	204,233
7	159	0.67	0.99	0.34	51.0	294,964
8	121	0.43	1.00	0.57	42.9	207,429
9	150	0.60	0.99	0.40	81.0	376,905
10	114	0.75	1.00	0.25	93.7	278,492
11	127	0.44	0.99	0.56	52.4	112,945
12	219	0.69	1.00	0.31	68.7	483,726
13	229	0.50	0.99	0.50	51.3	493,688
14	580	0.69	1.00	0.31	373.7	1,379,847
15	459	0.60	0.99	0.40	49.7	10,123
16	450	0.64	1.00	0.36	263.5	631,485
17	544	0.71	1.00	0.29	184.2	765,574

ED, emergency department; LR, likelihood ratio.

## 4 Discussion

To our knowledge, this is the first study to assess the diagnostic accuracy of ICD-based coding within 2 commonly used EMR systems for PE surveillance on a weekly and monthly basis. At both times, approximately one-half of the PE+ cases were correctly identified by the ICD I26 leader with false-positive rates at or <1% when compared against a rigorous criterion standard that used a combination of human adjudication and computer interpretation of CTPA reports. While some researchers may wish to have extremely low latency in data, requiring weekly reporting, we anticipated that workflow and systems issues could contribute to delay in appearance of ICD codes. For example, emergency clinicians may not complete charts from patients seen near the end of the week, precluding their ICD coding before Monday extraction. Moreover, the billing and coding professionals may require several days to enter the ICD codes. We found a potentially clinically important increase in sensitivity from weekly extraction (50.9%; 95% CI, 46.1%-55.6%) to monthly extraction (57.5%; 95% CI, 52.7%-62.1%), which can inform the content of future VTE surveillance protocols. We also believe this is the first study to examine interhospital variation in the diagnostic accuracy of ICD26 coding for detection of PE in the ED setting. This demonstrated a consistently high LR (+) across 17 sites. Similar reliability between hospitals has been reported for inpatient samples in the United Kingdom [26].

### 4.1 Comparison with previous studies and implications for policy

The sensitivity of the ICD26 leader code determined in our study is <73% pooled sensitivity estimate from 7 previous cohort studies [19]; by contrast, we observed higher specificity. One interpretation of our present findings is that the ICD I26 leader can produce a modest LR (−) but affords an extraordinarily high LR (+) and predictive value positive, allowing confidence that when I26 is located in the string of ICD codes, it is convincing evidence of a new or recurrent PE seen on CTPA by a board-certified radiologist. In other words, a large fraction of new and recurrent PE cases will be missed with ICD-based surveillance, but the false-positive rate is extremely low. Table 2 suggests that the specificity and LR (+) remains constant across sites, and the regression analysis suggests that larger hospitals may have more reliably high LR (+) results. The latter observation might be predicted because larger hospitals could be hypothesized to have more billing and coding resources. Our data imply that the I26 leader can be used to predict the total number of PE cases, estimated by multiplying the actual number detected times a coefficient (eg 1.4), with a statistically transparent margin of error, which would have to be determined by the variability in diagnostic sensitivity using a criterion standard such as CTPA reports. The high specificity, predictive value positive, and LR (+) also suggest that the ICD26 leader can be used to make inferences about risk between patient subgroups (eg, race or age), as well as geographical and time-varying changes such as season. Additionally, as shown in Table 1, detailed patient-level data can be downloaded for each case identified. The addition of the I82 ICD code for DVT did not substantially improve sensitivity. Moreover, in the hospitals studied, among the 1,431,519 patients with ARI, only 14 patients underwent VQ scanning within 30 days, suggesting that for any future US surveillance network, CTPA scanning comprises the diagnostic method of choice to survey for pulmonary vascular imaging-proven PE. These findings also show that a future surveillance network should strive to actively identify CPTA reports and use an automated system such as REGEX or other computerized program to complement the ICD26 leader in effort to identify most cases of PE in the ED.

### 4.2 Strengths and limitations

To our knowledge, this represents the first work to determine the potential accuracy of low-latency surveillance of PE, on a weekly or monthly basis, using ICD codes entered into the EMR at the time of patient exit from the ED. It should be recognized that this work differs from previous research into the accuracy of ICD coding because it was not a secondary analysis of an administrative database, but rather was a deliberately programed methodology, designed for real-time surveillance of patients with ARIs across a large sample of EDs representing all 11 Department of Human and Human Services health regions. A second novel aspect of our work was the use of READ-PE to crosscheck human interpretation of CTPA reports, which found that in our case, false-positive errors were introduced by an anomaly that sometimes occurred when the users closed the electronic survey instrument. This error would not have been detected without the READ-PE crosscheck. Thus, we believe that any future surveillance network would be best served by using a combination of ICD26 capture, supplemented by a computerized method, such as READ-PE or natural language processing, applied to all CTPA reports. The third novel finding, provided by the RESP-LENS network design, was the ability to test the sensitivity and specificity of the ICD26 leader across multiple hospitals. These data demonstrated that the LR (+) not only remained robust across sites but also showed preliminary inference that hospitals with smaller volumes are more likely to have lower predictive value of the ICD26.

Limitations to this work include biases inherent to restricting the sample to patients undergoing CTPA; we cannot extrapolate the findings to patients who did not undergo CTPA. This could introduce bias into the sample. However, it appears extremely unlikely that PE is being diagnosed with VQ scanning in the US, although this could be different in other countries. The addition of the I82 (DVT) ICD did not substantially improve detection. Another limitation is the lack of data to provide insight for the low sensitivity. Possible reasons include latency or human factor errors in ICD coding. Additionally, the data show the fallibility of using order entry as a method to locate CTPA, given that 13% were not actually CTPA scans. Lastly, we do not have data to indicate whether sensitivity for the I26 leader would increase over a longer time frame.

## 5 Conclusion

This work demonstrates for the first time, the potential utility and limitations of the ICD26 leader code for contemporaneous surveillance of PE. The ICD26 leader has high positive predictive value, evidenced by a pooled specificity of >99% that was stable across sites, but with a sensitivity of 57%. These data suggest the opportunity to use the ICD26 to surveil PE cases on a monthly basis from existing EMRs using the ICD26 in the ED setting.
