# Supplementary material for: Diagnostic accuracy of the international classification of disease “I26” code to detect acute pulmonary embolism in a surveillance network
Source: Res Pract Thromb Haemost. 2025 May 23;9(4):102900. doi: 10.1016/j.rpth.2025.102900 (PMC12205332; doi:10.1016/j.rpth.2025.102900)
Supplement: Supplementary Material [file mmc1.pdf]

Image Review

Record ID	__________________________________
RESP-LENS ID	__________________________________<br>(Created via Import from Studymaker File 2024-11-22)
Site ID derived from RESP-LENS record ID	__________________________________<br>(For auditing purposes)
Site Code	__________________________________
Does the narrative indicate that the examination was NOT a CT scan of the chest (e.g., not a CT or a CT of another body part)?<br><br>If yes, then stop and move on to next file.	◯ Yes<br>◯ No
Did the CT have IV contrast?	◯ Yes<br>◯ No
CT Findings	____
VTE Finding (Yes/No/Indeterminate)	◯ Yes<br>◯ No<br>◯ Indeterminate
VTE Positive associated findings	☐ Acute or no mention of findings of chronic PE<br>☐ The report contains a mention of chronic PE<br>☐ Mention of right heart strain (RV enlarged, contrast reflux) or "massive" PE<br>☐ Mention of bilateral PE<br>☐ Mention of saddle PE<br>☐ Mention of pulmonary infarction
Comment on pulmonary artery or pulmonary hypertension	◯ No mention of the pulmonary artery<br>◯ Report contains comment that the pulmonary artery is enlarged or other mention of pulmonary hypertension
Mention of indeterminate findings?	☐ No mention of inadequate or indeterminate features<br>☐ Report contains comment of inadequate visualization/opacification of the distal pulmonary arteries (may say "segmental or subsegmental")<br>☐ Report contains mention of motion artifact
Pneumonia Positive	◯ Yes<br>◯ No<br>◯ Indeterminate

Pneumonia other comments	☐ Use of words "consolidation", "dense", or "lobar"<br>☐ Mention of "bilateral" or "multilobar"<br>☐ Use of "interstitial" or "viral"<br>☐ Mention of a pleural effusion
Suspected cancer?	◯ Yes, the report mentions a finding suspicious or diagnostic of cancer<br>◯ No, no mention of cancer<br>◯ Indeterminate
This is a case I think Kline should see	◯ True<br>◯ False
Time Spent Reviewing (minutes)	__________________________________
Comments on Reviewing this Record	____
